# Hepcidin discriminates sepsis from other critical illness at admission to intensive care

**DOI:** 10.1038/s41598-022-18826-0

**Published:** 2022-09-01

**Authors:** Jon Olinder, Alex Börjesson, Jakob Norrman, Tobias West, Joakim Carlström, Alexander Gustafsson, Martin Annborn, Heiko Herwald, Cecilia Rydén

**Affiliations:** 1 Department of Infectious Diseases, Helsingborg Hospital, Helsingborg, Sweden; 2 Division of Infection Medicine, Department of Clinical Sciences, Lund University, Lund, Sweden; 3 Department of Internal Medicine, Helsingborg Hospital, Helsingborg, Sweden; 4 Department of Intensive Care Medicine, Helsingborg Hospital, Helsingborg, Sweden; 5 Present Address: Department of Psychiatry, Karlskrona, Sweden; 6 Present Address: Husläkarna Falkenberg, Falkenberg, Sweden; 7 Present Address: Hospital of Hässleholm, Hässleholm, Sweden; 8 Present Address: Malmö Addiction Center, Malmö, Sweden

**Keywords:** Biomarkers, Medical research

## Abstract

Initial differential diagnosis and prognosis for patients admitted to intensive care with suspected sepsis remain arduous. Hepcidin has emerged as a potential biomarker for sepsis. Here we report data on the relevance of levels of hepcidin versus other biomarkers as a diagnostic and prognostic tool for sepsis. 164 adult patients admitted to the intensive care unit (ICU) within 24 h upon arrival to the hospital were included. Blood samples collected daily for seven consecutive days and hepcidin levels, heparin binding protein (HBP) levels and standard biomarkers were determined. Blood cultures were initiated at inclusion. Clinical scores were evaluated daily and mortality after 28- and 180-days was recorded. One hundred of the patients were found to fulfil the criteria for sepsis whereas 64 did not. Hepcidin levels at admission were significantly higher in the septic than in the non-septic patients. In septic patients hepcidin levels declined significantly already at 24 h followed by a steady decline. A significant negative correlation was observed between hepcidin levels and SAPS 3 in patients with sepsis. Hepcidin levels at inclusion were significantly higher among septic patients that survived 180-days and predicted mortality. Our data show that hepcidin levels are indicative of sepsis in patients admitted to the ICU and has a prognostic value for mortality.

## Introduction

Sepsis, defined as multiorgan dysfunction due to a dysregulated host response to infection causes substantial morbidity and mortality with a negative impact on hospital and financial resources globally^1,2^. The severity of sepsis depends on several host factors, such as age, comorbidities, and immune status, as well as pathogen factors such as virulence, microbial species, and infectious load^1,3,4^. In spite of the sepsis-3 criteria presented as a consensus document 2016 the diagnosis is often a challenge in critically ill patients^5^. The global incidence of sepsis was estimated to almost 50 million cases in 2017 with a mortality rate of 22%, with the highest burden reported from sub-Saharan Africa, Oceania, and major parts of Asia, though sepsis mortality rates are falling in high income countries^6^. Sweden has an incidence of sepsis of 780/100 000 and one third decease within three months after ICU admission^7,8^.

To distinguish sepsis from other critical illness, clinicians largely have to rely on the Sequential Organ Failure Assessment (SOFA) score and on microbial identification. Additional biomarkers with high sensitivity and specificity may be beneficial for diagnosis and to monitor disease progression and assessment of the effect of antibiotics in bacteria-induced sepsis^9,10^. This would allow de-escalation of specific treatment in sepsis caused by bacterial infection, thereby minimizing antibiotic use and the risk for resistance development^9–11^.

Several potential biomarkers for monitoring sepsis have been investigated, including white blood cell count (WBC), C-reactive protein, (CRP), lactate, and procalcitonin (PCT). During the last decade Heparin binding protein (HBP) has attracted interest as a biomarker for severe bacterial infection including sepsis and meningitis^12,13^. HBP also known as Cationic Antimicrobial Protein of 37 kDa (CAP37) was identified in 1984 and is produced by neutrophils, stored in intracellular vesicles, and rapidly released upon stimulation by pathogen associated microbial patterns, PAMPs^12,14^. Elevated HBP levels have been shown to correlate with hypotension and organ dysfunction in patients with bacterial sepsis but failed to distinguish patients with septic shock from other causes of shock^15,16^.

More recently hepcidin, identified as a major regulator of iron metabolism, has emerged as a potential marker for bacterial sepsis^17–19^. Serum levels of hepcidin are increased both in children with severe infections and in adults with sepsis^18,20,21^. It has also been reported to be a marker of acute kidney injury in patients at the ICU^22^. In a pilot study we reported that hepcidin levels increased in septic shock patients, similar to PCT, and prior to increases in CRP levels during disease progression^23^. Hepcidin levels decreased within the first 24 h of admittance to the ICU.

Hepcidin is a cysteine-rich disulphide bonded 25 amino acid peptide identified in year 2000 as primarily a key regulator of iron homeostasis but also exhibiting antibacterial and antifungal activity^19,24^. Hepcidin is synthesized by hepatocytes and is upregulated by high serum iron levels^25^. Hepcidin acts as an early responding acute phase reactant regulated by IL-6 and in response to lipopolysaccharide^26–28^. Infusion of IL-6 in humans increases urine hepcidin levels and decreases serum iron and transferrin saturation within a few hours^27^.

The present study was undertaken to evaluate whether hepcidin levels in serum from newly admitted ICU patients could discriminate community acquired severe sepsis/septic shock from critical illnesses due to non-septic conditions. Furthermore, we investigated the potential value of hepcidin levels compared to HBP levels and the commonly used biomarkers for sepsis *i.e*. white blood count (WBC), C-reactive protein (CRP), lactate, and procalcitonin as prognostic markers of outcome of disease.

## Methods and patients

### Definitions and patient population

The study was conducted at the ICU at the tertiary hospital of Helsingborg, Sweden from May 2014 until August 2020, and did not include any patients with Sars-Cov-2. All methods in the manuscript were carried out in accordance with relevant guidelines and regulations.

Patients all above 18 years of age, who had to be admitted to the ICU within 24 h of arrival to the hospital and were assessed by Simplified Acute Physiology Score III (SAPS3) and daily Sequential Organ Failure Assessment (SOFA) criteria according to routine performance in the ICU^1,29^. Patients having a SOFA score ≥ 2 in need of intensive care for at least 3 days and initially in need of assisted ventilation and/or vasopressors during the ICU-stay were included in the study. Exclusion criteria included blood transfusion or surgery within 7 days preceding the current hospitalization and expected ICU care of ≤ 2 days. Results of hepcidin and HBP in fifteen of the patients with septic shock were part of the pilot study published in 2020^23^.

The study was approved by the Regional Ethics Committee of Scania county, Lund, Sweden nb: 2014/195 and 2015/467. Oral and written consent was collected either from the patient or next of kin, where the latter approved consent if the patient was medically unstable and thus not able to confirm upon inclusion, thus, delayed consent from the patient was accepted by the Ethics Committee.

### Data collection

Patient data collected at enrolment included demographics and co-morbidities. Length of stay (LOS) and any complications occurring at the ICU, the need of dialyses, fluid resuscitation, and temperature were recorded (Tables 1, 2). Blood samples were obtained at the time of inclusion and every morning during seven consecutive days. Blood cultures were performed upon admittance to the ICU on all patients. The primary organ dysfunction causing the enrolment was registered. Clinical evaluation was registered by SAPS 3 at admission and by daily SOFA score in the ICU. In hospital mortality, as well as 28- and 180-day mortality were assessed.

**Table 1:** Patient characteristics.

Patient group	Sepsis, n = 100 (61.0%)	Non-sepsis, n = 64 (39.0%)
Age, median (IQR)	69.5 (57.0–76.0)	70.0 (60.3–76.0)
Sex, male (%)	55 (55.0)	35 (54.7)
**Comorbidities, n (%)**		
Cardiovascular disease	48 (48.0)	26 (40.6)
Hypertension	31 (31.0)	26 (40.6)
Diabetes mellitus	25 (25.0)	18 (28.1)
COPD	14 (14.0)	17 (26.6)
Chronic kidney disease	12 (12.0)	5 (7.8)
Malignancy	10 (10.0)	5 (7.8)
Liver disease	6 (6.0)	0
**Source of infection, n (%)**		
Respiratory tract	41 (41.0)	–
Urogenital	20 (20.0)	–
Skin or soft tissue	17 (17.0)	–
Abdominal	11 (11.0)	–
Unknown	10 (10.0)	–
Endocarditis	1 (1.0)	–
**Non-infectious conditions, n (%)**		
Cardiovascular	–	22 (34.4)
Abdominal	–	12 (18.8)
Trauma	–	9 (14.1)
Neurological	–	7 (10.9)
Respiratory	–	7 (10.9)
Metabolic	–	3 (4.7)
Other	–	3 (4.7)
Renal	–	1 (1.6)
**ICU data**		
Intubation, n (%)	50 (50.0)	57 (89.1)
Non-Invasive Ventilation, n (%)	16 (16.0)	6 (9.4)
Fluid resuscitation day 1 , mL, median (IQR)	5962 (3186–7524)	4053 (1366–6053)
Vasopressors, n (%)	97 (97.0)	52 (81.3)
In need of dialysis during ICU stay, n (%)	21 (21.0)	13 (20.3)
Weight at admission, kg (IQR)	79.5 (67.5–94.5)	70.5 (49.0–87.0)
ICU stay ≥ 7 days, n (%)	34 (34.0)	16 (25.0)
Complications during ICU stay, n (%)	34 (34.0)	37 (57.8)

**Table 2:** Laboratory and clinical evaluation measurements at enrolment.

	Septic shock, n = 100	Non-sepsis, n = 64	*p *value	Adjusted *p *value
**Biomarkers at admission, median (IQR)**				
Hepcidin (nmol/L)	41.0 (21.0–66.0)	11.5 (3.9–35.5)	< 0.001	< 0.001
HBP (ng/mL)	44.0 (27.5–90.0)	20.6 (13.5–39.4)	< 0.001	< 0.001
CRP (mg/L)	180.0 (113.0–296.0)	31 (8.9–103.0)	< 0.001	< 0.001
PCT (µg/L)	33.00 (6.6–100.8)	1.05 (0.22–5.28)	< 0.001	< 0.001
Lactate (mmol/L)	2.8 (1.5–4.4)	2.2 (1.1–3.8)	0.104	0.94
WBC (× 10^9^/L)	11.8 (8.0–19.1)	11.3 (7.0–16.5)	0.620	1.00
**Clinical parameters at admission, median (IQR)**				
Body temperature	37.4 (36.6–38.5)	36.4 (35.7–36.9)	< 0.001	< 0.001
SOFA-score	11.0 (7.0–12.0)	9 (5.3–10.0)	0.007	0.063
SAPS III score	68.0 (60.0–75.0)	65.0 (53.0–79.0)	0.337	1.00
**Cultures, n (%)**				
Gram positive	37 (37.0)	–		
Gram negative	23 (23.0)	1 (1.6)		
Multiple findings	9 (9.0)	1 (1.6)		
Viral findings	3 (3.0)	–		
Negative cultures	22 (22.0)	45 (70.3)		
Contamination	3 (3.0)	–		
No cultures withdrawn	3 (3.0)	13 (20.3)		

Bonferroni corrected *p* value for 9 comparisons.

### Laboratory methods

Hepcidin concentration was determined by Mass Spectrometry using a 6500 Q-Trap®, Sciex, Washington D.C., USA, at the Clinical Chemistry Laboratory at Lund University Hospital, Sweden^30^. Heparin binding protein analyses were performed with an in-house developed sandwich enzyme-linked immunosorbent assay (ELISA) at the Department of Clinical Science, Lund University, Sweden, as previously described^31^. Analyses of white blood count (WBC), CRP, PCT, lactate, and all other routine blood chemistry analyses as well as rapid diagnostic tests for virus and certain bacteria were performed at the Clinical Chemistry Laboratory, Helsingborg Hospital, Sweden. Antigen tests for group A streptococci, as well as urine tests for *Legionella* and pneumococcal antigens were performed when appropriate. Microbial analyses were performed at the Clinical microbiological laboratory at Lund University Hospital, Sweden, including blood cultures as well as cultures from other adequate locations *e.g.* wounds, urine, sputum, and nasopharynx, were obtained at inclusion and repeated according to the progression of the patients’ illness. PCR analyses were performed when viral infections were suspected.

#### Hepcidin and HBP concentrations

Hepcidin levels were determined by mass-spectrometry at the the Clinical Chemistry Laboratory, Lund University Hospital. Hepcidin levels above 12 nmol/L were considered elevated according to the normal reference values of hepcidin that are set to 1–12 nmol/L in adults according to the local Reference Laboratory, in line with previously reported values^32,33^. A similar interval was used by Schoorl et al*.* in their study on infected patients versus a control group^34^. Concentrations of HBP were determined by an in-house ELISA method and levels ≥ 20 ng/mL were considered elevated^15,31^. This is in accordance with levels of HBP measuring > 15–30 ng/mL having high prognostic value on the development and outcome in patients with sepsis and/or septic shock^7,15,35^.

### Statistics

Statistical work was conducted using IBM SPSS Statistics 26 (IBM, Armonk, New York, US). All statistical analyses were performed at the final stage of the study when patients had been dichotomized into sepsis according to Sepsis-3 criteria determined during the treatment period and non-sepsis, which was unknown at admission. Since data from the biomarkers were not normally distributed, primarily non-parametric tests were performed. Median and interquartile ranges (IQR) were reported as appropriate. When performing, ROC-curves, Spearman’s signed rank test and logistic regression models, a confidence level of 95% was used and a *p *value < 0.05 was considered statistically significant.

Mann Whitney U-test was performed when comparing arrival data of biomarkers, temperature, and clinical scores between the sepsis and the non-sepsis groups. Due to multiple comparisons, the Bonferroni correction method was used.

The dynamics of the biomarkers, including hepcidin, HBP, CRP, PCT, lactate, WBC, haemoglobin, and creatinine, were illustrated as clustered box plots for both septic—and non-septic patients. Values registered at time zero represented baseline inclusion values from blood samples drawn at the ICU.

A receiver operating characteristics (ROC) curve including hepcidin, HBP, CRP, PCT, serum lactate, and WBC was performed to assess the diagnostic power for septic shock of each individual biomarker. Sensitivities, specificities, positive predictive values, and negative predictive values were calculated from cross-tabulations. Furthermore, a logistic regression was performed to analyse the ROC-curves combining two biomarkers, in this case hepcidin and HBP together with PCT and CRP respectively. Wilcoxon´s signed rank test was used to illustrate how fast the biomarkers were reduced in relation to inclusion values. Median values of hepcidin, HBP, CRP, PCT, lactate, WBC, SAPS3, SOFA-score, and body temperature were analysed at admission among survivors and non-survivors at a 180-day mortality follow-up. Mann Whitney U-test was performed to analyse possible correlations between admission values, levels of biomarkers, clinical evaluation tools (SAPS III, SOFA score, and body temperature) and survival at 180-days.

A binary logistic regression model with an interaction effect was utilized to investigate if admission values of hepcidin and HBP could predict 28- and 180-days mortality in the respective patient group. The dependent variable was set to 28- and 180 days mortality. The interaction effect was set as the effect of admission values of hepcidin and HBP (time 0), respectively, representing the independent variable in the sepsis group versus the non-sepsis group compared to all survivors in the cohort. Time 0 was chosen since the variability of biomarkers later during the ICU stay could be influenced *e.g.* by treatment, and secondary infection influencing the statistical analysis.

Spearman’s signed rank correlation coefficient was used to analyse the correlation between biomarkers and SOFA-score and SAPS 3, the latter only determined at admittance.

### Ethical approval and consent to participate

This study was approved by the Regional Ethics Committee of Scania county, Lund, Sweden nb: 2014/195 and 2015/467. Subjects provided written informed consent.

## Results

### Patient characteristics

**Figure 1:**
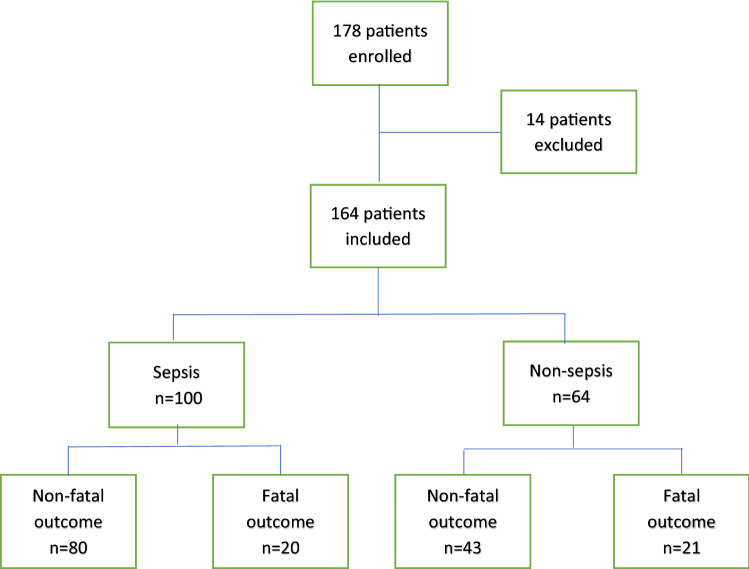
Patient flowchart. Fatal outcome equivalent to 28 days mortality. At 180 days, an additional four patients deceased in each group, respectively.

A total of 164 out of 178 patients admitted to the ICU within 24 h upon arrival to the hospital were included into the study. At the evaluation of the study, 100 of these patients fulfilled criteria for community acquired severe sepsis/septic shock, (97 according to the sepsis-3 criteria for septic shock and three patients included prior to 2016 according to sepsis-2 criteria with severe sepsis with organ dysfunction^1,36^ and were defined as the sepsis group) (Table 1). The non-sepsis group consisted of 64 patients with non-septic conditions, requiring ICU-care with assisted ventilation and/or vasopressor support. This prospective, observational study over seven consecutive days did not include any control group of subjects. The additional fourteen patients initially included, eight with septic shock and six non-septic patients had to be excluded from the final cohort due to lack of data (Fig. 1). The gender distribution and median age in the two groups were similar, whereas comorbidities differed between the groups (Table 1). The most common organ system affected was the respiratory tract in septic patients (41.0%) and the cardiovascular system in one third of the non-sepsis patients (Table 1).

Intubation and non-invasive ventilation were required more frequently in the non-sepsis group of patients (Table 1). In the sepsis group, larger fluid volumes administered during the first 24 h, vasopressor support, and a longer stay in the ICU were more common than in the non-sepsis group of patients. Cardiac complications, seizures, ventilator-assisted pneumoniae, and pleural effusions affected the non-septic patients more frequently than the sepsis patients, whereas the need for dialysis was similar between the groups (Table 1).

### Microbiological data

Blood cultures were performed at admittance to the ICU to be able to evaluate if the critical illness could be due to sepsis or not and were drawn in 148/164 of the patients. Eventually 97/100 of the patient finally diagnosed with sepsis had blood cultures performed and 51/64 patients in the non-septic patients (Table 2). Positive bacterial cultures were found in 69/100 (69.0%) of patients with sepsis and 48 of the sepsis patients had clinically relevant positive blood cultures, whereas other diagnostic tools *i.e.* positive pneumococcal antigen in urine (4/100), positive rapid antigen test for group A streptococcus from tissue (4/100), and the remaining positive cultures were assessed as relevant from tissue or sputum cultures (13/100). Gram positive bacteria dominated in single culture findings and *Escherichia coli* was the most commonly isolated Gram-negative bacterium (Supplementary information, Fig. 1). Two patients were positive for influenza and one patient had a respiratory syncytial virus infection. None of the patients were found to suffer from community acquired fungal or parasitic infection. In the non-sepsis group blood cultures were performed in the vast majority of patients. All patients with septic shock received broad-spectrum antibiotics according to local treatment guidelines after adequate blood cultures had been obtained. The body temperature at admission was significantly higher in septic than in non-septic patients.

### Admission levels and time dependant changes of biomarkers

**Figure 2:**
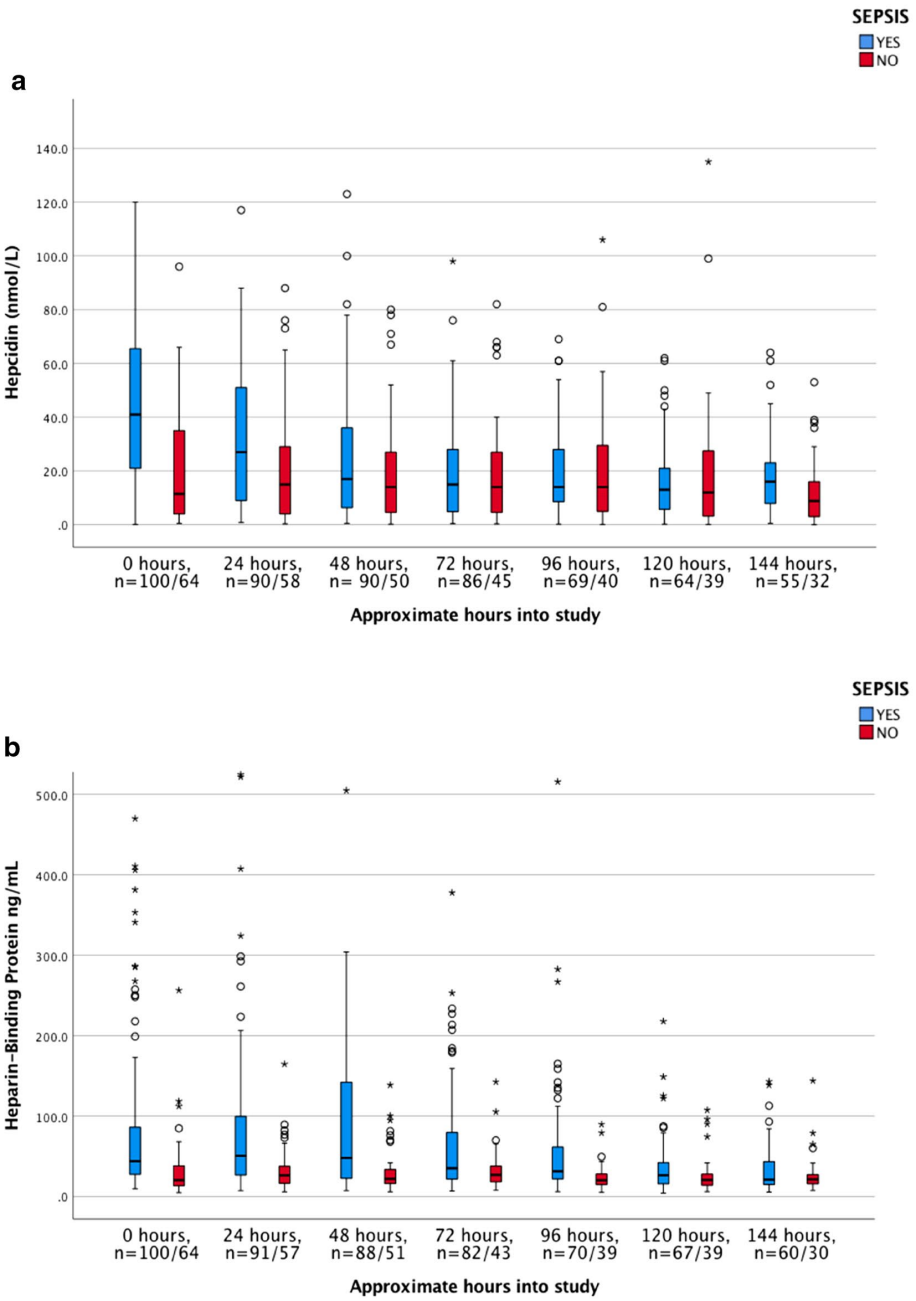

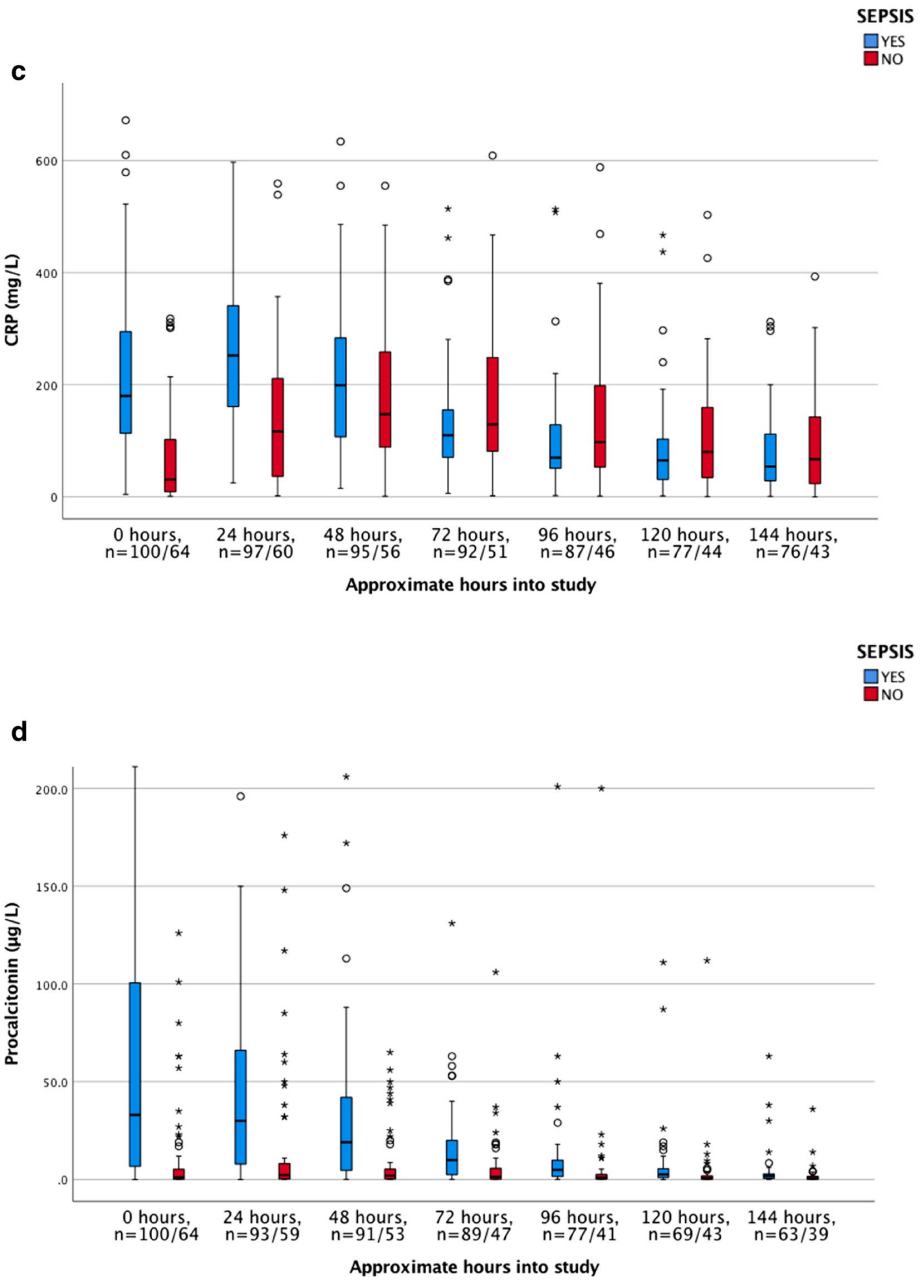

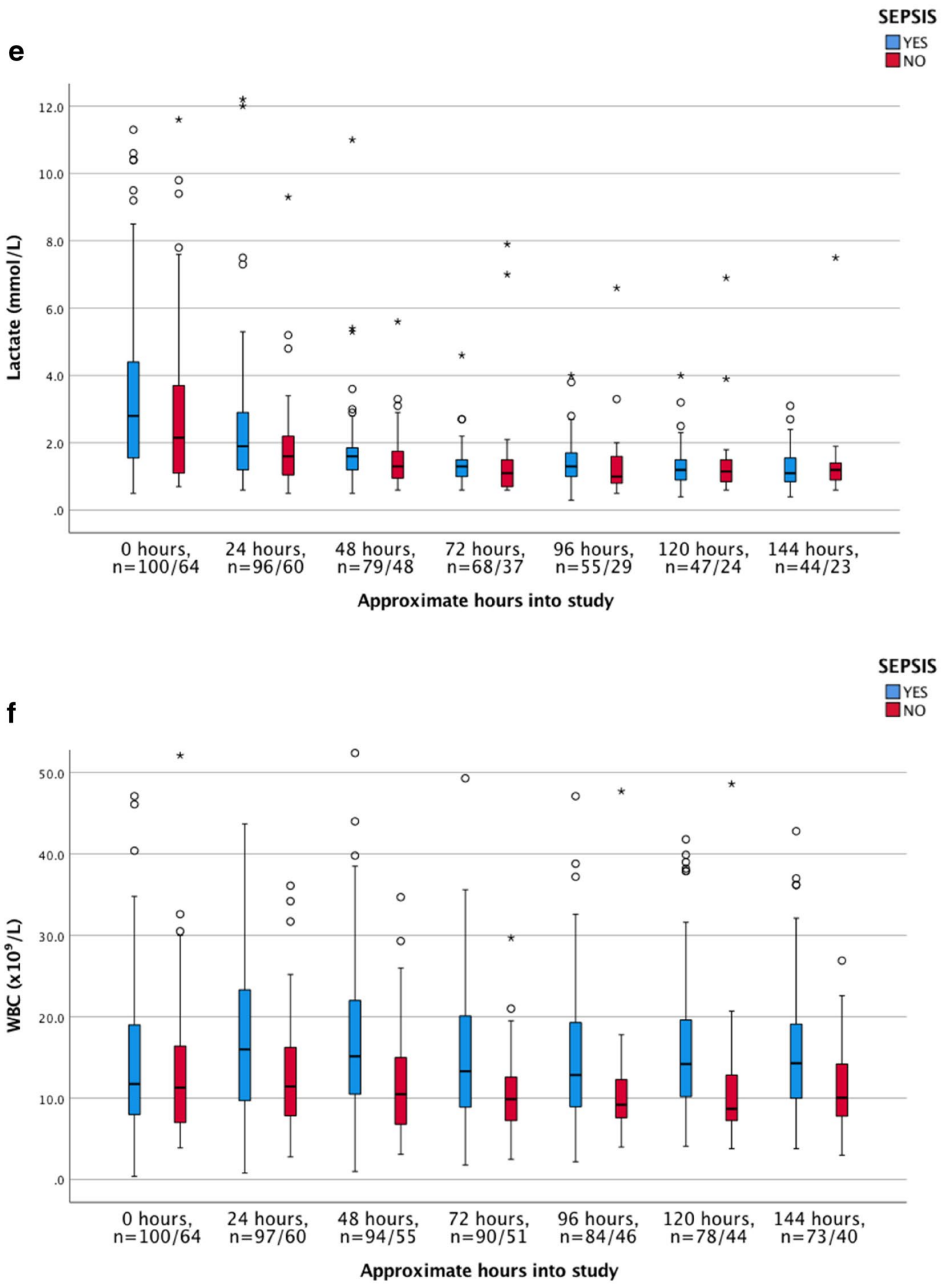
(**a**–**f**) Dynamics of biomarkers during the study period. Boxplot. [hepcidin, HBP, CRP, PCT, lactate and white blood count (WBC). Due to de-escalation of y-axis a few extreme- and ordinary outliers are not visualized in Fig. 3a–f. n = numbers, sepsis versus non-sepsis, respectively. Missing data occurred occasionally at single time points distributed between both septic and non-septic patients.

Admission levels of analysed biomarkers are shown in Table 2. As previously mentioned, the normal reference value for hepcidin are set to 1–12 nmol/L in adults according to the local Reference Laboratory, based on previous reports conducted from age- and sex-stratified samples from the general population^32^. Hepcidin values at admission were significantly higher in the sepsis than in the non-sepsis group of patients, 41.0 nmol/L (IQR 21.0–66.0) versus 11.5 nmol/L (IQR 3.9–35.5), (*p* < 0.001), with the highest concentrations recorded already at admission. Hepcidin levels were higher in septic patients with than without positive cultures 55.0 versus 21.0 nmol/L (Data not shown). Concentrations of biomarkers were analyzed for seven consecutive days, although with some missing values that varied between the biomarkers (Fig. 2a–f). Hepcidin levels decreased in all sepsis patients during the first 24 h after admittance and continued to decrease during the 7-day study period (Fig. 2a). This decrease was paralleled by a significant decrease in PCT and lactate levels (Fig. 2d,e, and Table 3, Wilcoxon´s signed rank test). Since there was a difference in numbers of the analyzed biomarkers (Fig. 2a–f) Wilcoxon´s signed rank test was only utilized for analyzing changes during the first 72 h (Table 3). Hepcidin levels in sepsis patients declined rapidly during the first treatment days (Fig. 2a).

**Table 3:** Change in biomarker levels in relation to arrival values.

Biomarker	Admission day	24 h	48 h	72 h
Hepcidin nmol/L (*p*)	41.0	27.0 (< 0.001)	17.0 (< 0.001)	15.0 (< 0.001)
HBP, ng/mL (*p*)	44.0	50.8 (0.221)	48.1 (0.671)	35.3 (0.025)
PCT, µg/L (*p*)	33.0	30.0 (0.036)	19.0 (< 0.001)	9.9 (< 0.001)
CRP, mg/L (*p*)	180.0	252.0 (< 0.001)	199.0 (0.193)	110.0 (< 0.001)
Lactate, mmol/L (*p*)	2.8	1.9 (< 0.001)	1.6 (< 0.001)	1.3 (< 0.001)
WBC, × 10^9^/L (*p*)	11.8	16.0 (0.002)	15.2 (0.027)	13.3 (0.670)

Median values of biomarkers during the first 72 h in the sepsis group. *P* values from Wilcoxon’s signed rank test, presenting if there is any significant change in biomarker levels between current value and arrival value. A *p* value < 0.05 is considered significant.

No significant daily variations and no increase of median hepcidin levels during the study period was observed in the non-septic patients (Fig. 2a).

HBP was elevated at admission in sepsis patients compared to non-sepsis patients, 44.0 ng/mL versus 20.6 ng/mL (*p* < 0.001). In contrast to hepcidin, HBP levels increased in septic patients to reach maximal levels 24 h slowly declining after admission with no significant decrease of HBP until 72 h after admittance to the ICU (Fig. 2b, Table 3). HBP levels were higher in septic patients with positive blood cultures, 47.0 ng/mL versus negative cultures 40.3 ng/mL (Data not shown). In the non-sepsis group median HBP was below reference range at admission, with non-significant daily variations during the whole study period (Fig. 2b).

Significantly higher concentrations of CRP and PCT were found in the sepsis than in the non-sepsis group at admission, with peak values of PCT noted at admission whereas CRP and WBC increased significantly during the first 24 h among the septic patients (Fig. 2a–f, Table 3). In the non-septic patients increasing levels of CRP were noted during the first 48 h after admittance whereas PCT levels were low during the whole study period (Fig. 2c,d).

In sepsis patients with and without bacteraemia the median admission values of CRP were 196.0 and 150.0 mg/L, respectively, with corresponding PCT levels 43.0 versus 10.0 μg/L, and WBC 11.0 versus 16.7 × 10^9^/L. There were no differences in lactate levels between sepsis patients with or without bacteraemia, 2.8 mmol/L (Data not shown).

As for the prediction of septic shock, hepcidin showed higher sensitivity, specificity, and positive and negative predictive value in diagnosing septic shock compared to HBP, although inferior to CRP and PCT (Supplementary information Fig. 1), but the negative predictive value for hepcidin was higher than for PCT.

**Figure 3:**
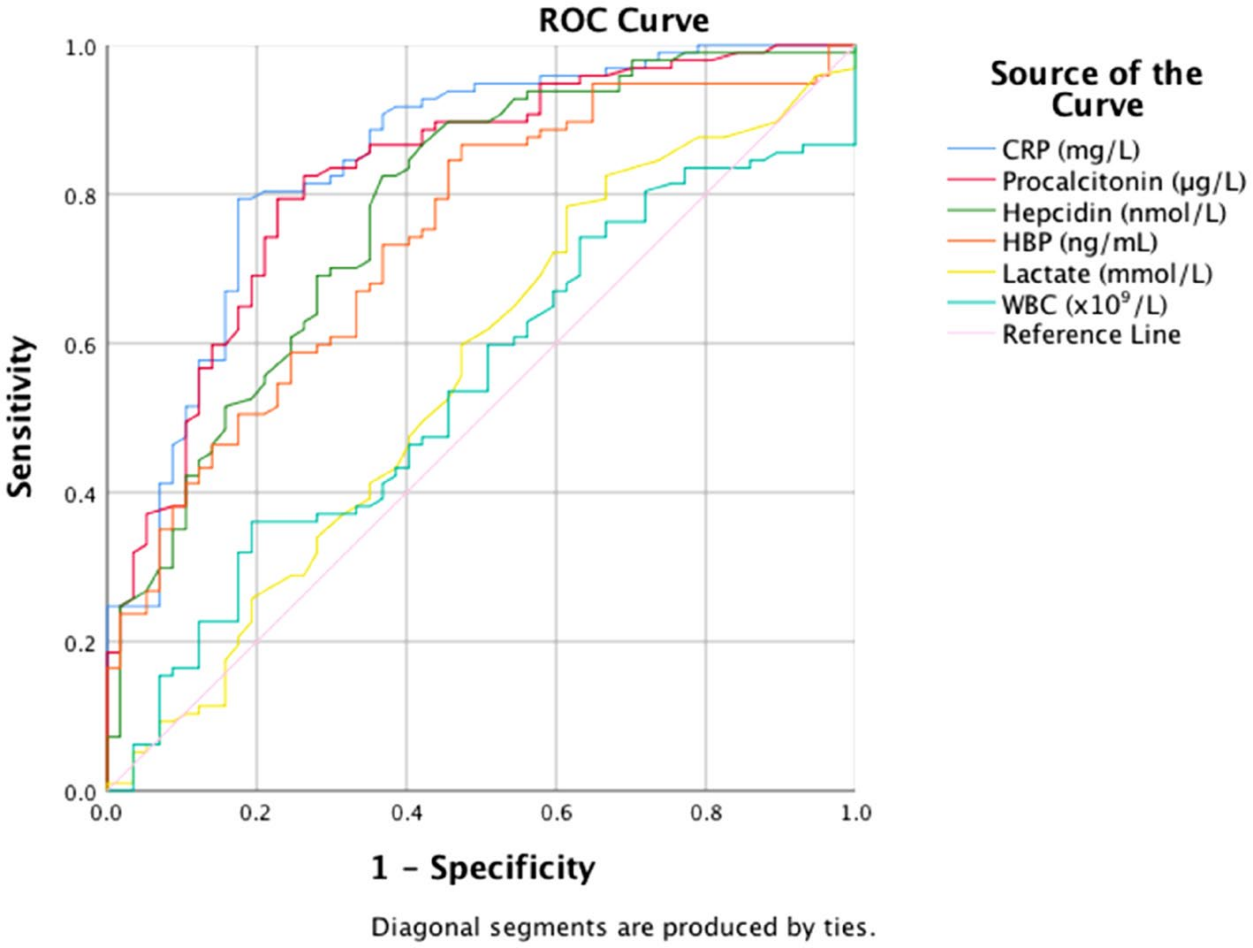
Receiver-operating characteristics curves of C-reactive protein (CRP), procalcitonin, hepcidin, heparin-binding protein (HBP), lactate and white blood cell (WBC) predicting sepsis/septic shock diagnosis.

CRP was superior to the other biomarkers as analysed with ROC curves with an AUC of 0.844, followed by PCT (0.825), hepcidin (0.780), and HBP (0.742), although all curves were highly significant (*p* < 0.001), whereas lactate (0.588) and WBC (0.541) were not useful in predicting septic shock (Fig. 3, Table 4).

**Table 4:** Areas under the receiver-operating characteristics curves presented of C-reactive protein (CRP), procalcitonin, hepcidin, heparin-binding protein (HBP), lactate and white blood cell (WBC).

Biomarker	AUROC (95% CI)	*p* value
CRP, mg/L	0.844 (0.779–0.910)	< 0.001
PCT, µg/L	0.825 (0.757–0.893)	< 0.001
Hepcidin nmol/L	0.780 (0.704–0.856)	< 0.001
HBP, ng/mL	0.742 (0.662–0.821)	< 0.001
Lactate, mmol/L	0.558 (0.462–0.654)	0.231
WBC, × 10^9^/L	0.541 (0.448–0.634)	0.393

**Figure 4:**
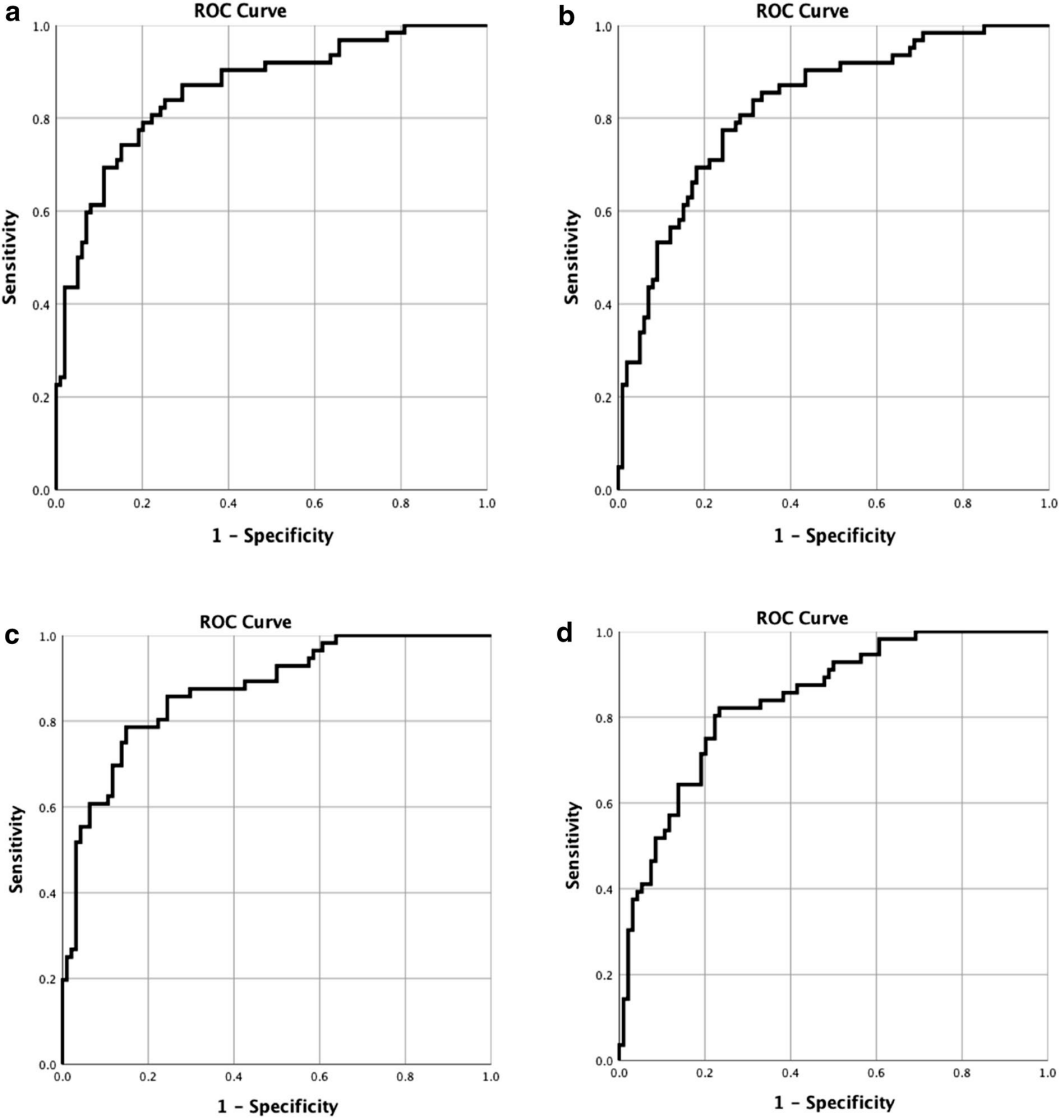
(**a**) ROC-curve with a combination of hepcidin and CRP at admission after performing logistic regression. AUC 0.861. (**b**) ROC-curve with a combination of hepcidin and PCT at admission after performing logistic regression. AUC 0.825. (**c**) ROC-curve with a combination of HBP and CRP at admission after performing logistic regression. AUC 0.872. (**d**) ROC-curve with a combination of HBP and PCT at admission after performing logistic regression. AUC 0.839.

Combinations of biomarkers used in this study were investigated by logistic regression. Thereby comparisons of AUC curves for hepcidin and HBP were analysed in combination with CRP and PCT respectively, (Fig. 4a–d). Logistic regression analysis revealed that hepcidin in combination with CRP reached an AUC of 0.861 (Fig. 4a) and HBP in combination with CRP reached an AUC of 0.872 (Fig. 4c). Hepcidin levels in combination with PCT reached an AUC of 0.825 (Fig. 4b), and for HBP in combination with PCT 0.839 (Fig. 4d).

### Biomarkers and clinical scores at admission

The median SAPS-3 scores between the groups did not differ significantly, whereas patients in the sepsis group presented higher SOFA score day 1 (Table 2). A negative correlation was observed between hepcidin and SAPS-3 for patients with sepsis, with a correlation coefficient of − 0.308 (*p* = 0.002) (Table 5). Lactate levels were associated with SAPS-3 at admission in both sepsis and non-sepsis patients (Tables 5, 6), in contrast to HBP, CRP, PCT, and WBC levels (Tables 5, 6). The association between the SOFA score and HBP levels showed a correlation coefficient of 0.286 (*p* = 0.005) in the sepsis group. Correlations were found at admission in the sepsis group between SOFA score and PCT and lactate levels, respectively, whereas no associations were observed between admission SOFA-score and hepcidin, CRP, or WBC (Table 5). In the non-sepsis group of patients, none of the biomarkers correlated significantly with SOFA score at admission (Table 6).

**Table 5:** Sequential Organ Failure Assessment (SOFA)-score and Simplified acute physiology score III (SAPS3) at admission analysed through Spearman’s rank correlation coefficient in the sepsis/septic shock group. Hepcidin*,* heparin-binding protein (HBP), procalcitonin (PCT), C-reactive protein (CRP), lactate and white blood cells (WBC).

Biomarker	SOFA-score at admission		SAPS3	
	*p* value	Correlation coefficient	*p* value	Correlation coefficient
Hepcidin	0.897	0.013	0.002	− 0.308
HBP	0.005	0.286	0.380	0.090
PCT	0.008	0.271	0.519	− 0.065
CRP	0.751	− 0.033	0.550	− 0.060
Lactate	< 0.001	0.599	0.001	0.326
WBC	0.199	0.132	0.308	0.103

**Table 6:** Sequential Organ Failure Assessment (SOFA)-score and Simplified acute physiology score III (SAPS3) at admission analysed through Spearman’s rank correlation coefficient in the non-sepsis group. Hepcidin*,* heparin-binding protein (HBP), procalcitonin (PCT), C-reactive protein (CRP), lactate and white blood cells (WBC).

Biomarker	SOFA-score at admission		SAPS3	
	*p *value	Correlation coefficient	*p* value	Correlation coefficient
Hepcidin	0.175	− 0.174	0.113	− 0.205
HBP	0.332	0.129	0.275	0.145
PCT	0.489	0.088	0.308	0.130
CRP	0.502	− 0.085	0.526	− 0.081
Lactate	0.104	0.205	0.005	0.349
WBC	0.927	0.012	0.271	0.141

#### Survival related to biomarkers and clinical scores

Ten of the 100 patients in the sepsis group and 13/64 in the non-sepsis group died within the first seven days after admittance to the ICU. At the 28- and 180-day follow ups, the mortality was 20.0% and 24.0% respectively in the sepsis group, versus, 32.8% and 39.1% in the non-sepsis group.

The median values at admission of biomarkers and clinical scores were compared between survivors and diseased at the 180-day follow-up for the whole group (n = 164), (Table 7). Hepcidin values were significantly higher at admission among survivors than diseased (37.0 nmol vs. 14.0 nmol/L, adjusted *p* < 0.001). Admission values of HBP, CRP, PCT, lactate, and WBC were not significantly different between survivors and diseased regarding 180-day mortality after Bon-Ferroni adjustment (Table 7), but without this adjustment CRP and PCT were significantly higher in patients that survived compared to the diseased, whereas lactate levels were significantly higher in patients that diseased. Among non-survivors SAPS 3, and SOFA score day 1 were significantly higher at admission compared to survivors at 180-days (Table 7).

**Table 7:** Biomarker and clinical evaluation levels at admission among survivors and diseased at 180-days for the whole group (sepsis and non-sepsis). Mann U Whitney test. (In the septic shock group, 24/100 patients had deceased at 180-days and in the non-sepsis group, 25/64 patients had deceased at 180-days). Bonferroni corrected *p* value for 9 comparisons.

Biomarker	Survivors (n = 115)	Deceased (n = 49)	*p* value	Adjusted *p* value
Hepcidin, nmol/L (IQR)	37.0 (16.0–64.0)	14.0 (5.0–40.0)	< 0.001	< 0.001
HBP, ng/mL (IQR)	34.8 (29.3–85.6)	38.2 (20.6–83.3)	0.467	1.00
CRP, mg/L (IQR)	142.0 (116.8–342.0)	100.0 (19.5–153.0)	0.013	0.117
PCT, µg/L (IQR)	18.0 (1.2–68.0)	3.6 (0.8–30.5)	0.046	0.414
Lactate, mmol/L (IQR)	2.2 (1.2–3.7)	3.3 (1.6–7.0)	0.011	0.099
WBC, × 10^9^/L (IQR)	11.2 (7.5–18.5)	13.0 (9.2–19.2)	0.361	1.00
SOFA (IQR)	9.0 (6.0–11.0)	11.0 (9.0–13.0)	0.001	0.009
SAPS 3 (IQR)	62.0 (54.8–70.0)	79.0 (71.0–84.0)	< 0.001	< 0.001
Body temperature	37.0 (36.4–38.4)	36.5 (35.7–37.5)	0.003	0.027

Hepcidin levels at inclusion correlated with 180-day mortality in the sepsis group (Table 9), but not with 28-day mortality (Table 8). Among septic patients, CRP was associated with 28-day mortality but not with 180-day mortality. Lactate, SAPS3 and SOFA score correlated both with 28- and 180-day mortality in the sepsis group, but no such associations were seen between PCT or WBC levels and mortality (Tables 8, 9). In the non-sepsis group, no association was noted between hepcidin levels and 28- or 180-day mortality (Tables 8, 9). Concentrations of HBP were not associated with 28- or 180-day mortality in the sepsis- nor in the non-sepsis group. In the non-sepsis group, the levels of CRP correlated with 28-day mortality. (Tables 8, 9). Lactate, SAPS3 and SOFA scores were higher among the non-survivors compared to survivors in the non-sepsis group and they were all associated with mortality at 28- and 180-days (Tables 7, 8, 9).

**Table 8:** Results from logistic regressions where 28-day mortality is set as the dependent variable, predicted by arrival values of biomarkers, SOFA or SAPS 3. An interaction model was used to account for different effects in the Sepsis/Non-Sepsis group. Odds ratio (OR) presented with 95% confidence interval. *CRP* C-reactive protein, *HBP* heparin-binding protein, *PCT* procalcitonin, *WBC* white blood cells, *SOFA* Sequential Organ Failure Assessment, *SAPS3* Simplified acute physiology score III.

Predictor	Sepsis		Non-sepsis	
	*p* value	OR (95% CI)	*p* value	OR (95% CI)
Hepcidin	0.147	0.987 (0.969–1.005)	0.470	0.990 (0.965–1.017)
HBP	0.501	1.001 (0.999–1.003)	0.580	1.001 (0.999–1.003)
PCT	0.828	0.999 (0.995–1.004)	0.753	1.001 (0.995–1.006)
CRP	0.017	0.995 (0.991–0.999)	0.034	0.996 (0.992–1.000)
Lactate	< 0.001	1.289 (1.124–1.479)	< 0.001	1.279 (1.119–1.463)
WBC	0.362	1.015 (0.983–1.049)	0.572	1.010 (0.977–1.044)
SOFA	< 0.001	1.272 (1.121–1.445)	< 0.001	1.283 (1.129–1.457)
SAPS3	< 0.001	1.132 (1.083–1.183)	< 0.001	1.132 (1.083–1.182)

**Table 9:** Results from logistic regressions where 180-day mortality is set as the dependent variable, predicted by arrival values of biomarkers, SOFA or SAPS. Odds ratio (OR) presented with 95% confidence interval. *CRP* C-reactive protein, *HBP* heparin-binding protein, *PCT* procalcitonin, *WBC* white blood cells, *SOFA* Sequential Organ Failure Assessment, *SAPS3* Simplified acute physiology score III.

Predictor	Sepsis		Non-sepsis	
	*p *value	OR (95% CI)	*p* value	OR (95% CI)
Hepcidin	0.044	0.981 (0.964–0.999)	0.169	0.981 (0.955–1.008)
HBP	0.682	1.000 (0.998–1.002)	0.798	1.000 (0.998–1.002)
PCT	0.800	0.999 (0.995–1.004)	0.645	1.001 (0.996–1.006)
CRP	0.067	0.997 (0.994–1.000)	0.151	0.998 (0.994–1.001)
Lactate	0.001	1.246 (1.092–1.432)	0.001	1.248 (1.095–1.423)
WBC	0.350	1.015 (0.983–1.049)	0.636	1.008 (0.976–1.041)
SOFA	0.001	1.219 (1.086–1.368)	< 0.001	1.237 (1.101–1.389)
SAPS3	< 0.001	1.112 (1.070–1.155)	< 0.001	1.112 (1.070–1.156)

## Discussion

The present study shows that serum hepcidin levels discriminate community acquired sepsis from other critical conditions requiring intensive care in a cohort of patients who were referred to intensive care within 24 h upon arrival to the hospital. The study followed the proposed standards for sepsis studies as proposed by Pierrakos et al*.* with the exception of a control group of patients or healthy individuals^37^. The study was unbiased since the admitted critically ill patients were subject to identical sampling and evaluation at the end of the study. Blood cultures were performed in the vast majority, 148/164, of all patients included. Hepcidin levels recorded at admission to the hospital were higher in those patients who later could be confirmed to be septic. Furthermore, hepcidin levels in septic patients declined rapidly during the following days in the ICU in contrast to the low but unchanged hepcidin levels recorded in non-septic patients with similar illness scores. Elevated levels of hepcidin at admission correlated positively with survival at 180 days.

The baseline characteristics in the patient cohort were comparable between the septic and the non-septic patients considering age, sex, and comorbidities with an overall distribution comparable to previous studies on patients treated in the ICU^38,39^. In the present cohort, septic patients had similar SAPS III but higher SOFA-score at admission than the non-septic patients and the need of vasopressor-support was higher. However, the non-sepsis group needed more invasive respiratory support, and both the 28-day and 180-day mortality rates were higher in the non-sepsis group.

Since the discovery of hepcidin in 2000 most published reports investigated the role of hepcidin in anemia, but also in critically ill adult patients including infectious diseases such as sepsis^17,18,40,41^. The studies by Wakakuri et al*.* and van Eijk et al*.* investigated patients admitted to a General Medicine Ward who fulfilled two criteria for systemic inflammatory response syndrome (SIRS), though they were not diagnosed in accordance with the currently used international criteria for septic shock^1,17,18^. These studies reported that hepcidin levels in serum were raised in sepsis patients. In a study including pediatric patients three groups of children were represented, 44 with sepsis and septic shock, 17 children treated in the ICU with no infection and 28 healthy children, reported that PCT and hepcidin levels were superior to discriminate between infected and non-infected children than CRP and WBC^42^. In a study by Yan et al*.* on 123 febrile children significantly higher concentrations of hepcidin in serum were recorded in the 11 children who had confirmed infections with pathogenic bacteria^43^. The general conclusions in the present study, namely that patients admitted to the ICU within 24 h of hospitalization, are consistent with the results in these previous studies, *i.e.* that patients afflicted by bacterial infections have increased serum levels of hepcidin in contrast to non-infected patients. Our study shows that hepcidin measurements in patients that had been admitted to the ICU within the first 24 h after hospitalization can improve differential diagnostic efficiency between septic and non-septic critically ill patients.

Antibiotic treatment was initiated already at admission and was adequate in the vast majority (98/100) of the septic patients. The recorded decrease in hepcidin levels with time may therefore reflect a reduction in bacterial load. This implies that the dynamics of hepcidin levels could reflect treatment efficiency at an early stage of treatment.

Both HBP and CRP concentrations increased during the first 24 h after admittance making the evaluation of these biomarkers less valuable at the early stage of disease. Hepcidin as well as CRP are known to be upregulated by IL-6 but data are to our knowledge not available on the finetuning and possible feed-back loops on IL-6 elicited signaling resulting in hepcidin versus CRP that could explain the results of the present study. In a report on surgical critical care patients Cherry-Bukowiec et al. reported that levels of CRP and IL-6 correlated with hepcidin and high CRP concentrations correlated with high same day hepcidin. Highest levels of hepcidin were noted on admission, whereas the highest CRP levels were noted after 24 h in accordance with our findings^44^. The temporal association with elevated hepcidin levels were similar to previous results presented in healthy individuals after lipopolysaccharide injection^28^ as well as in surgical patients in the ICU^44^. Our results showed that hepcidin peaks earlier compared to CRP, suggesting that hepcidin might be more relevant to analyse in the acute setting (< 24 h) of inflammation, also in line with previous reported data^44^. Hepcidin analysis could add value at an early phase of disease to the commonly used lab analyses in support of a septic cause of disease. Hepcidin could indicate which patients are at risk for a more severe course of illness if several inflammatory markers with different origins of synthesis are affected *e.g.* CRP and hepcidin.

Logistic regression analysis of receiver operating characteristic (ROC) curves for levels of HBP in combination with CRP gave an AUC of 0.872, and an AUC of 0.861 for hepcidin in spite of that CRP levels increased in both septic and non-septic patients during the first 24 h at the ICU. Our data support earlier findings on increased HBP in septic shock^15^, though our present data show that increased HBP levels persisted for a longer time period during the ICU care than what were reported in the previous studies^15,45,46^. We recorded low levels of HBP in the non-septic patients further supporting a non-septic cause of critical illness. This agrees with earlier findings by Chew et al. and Bergquist et al*.*^16,47^. Increased HBP levels have been reported not only in septic shock but also associated with both circulatory and respiratory failure in critically ill patients^45,46^. In summary HBP added information on patients regarding septic illness but as judged from the present study secondary to hepcidin.

We noted a significant association between hepcidin in the septic shock patients as a predictor of 180-day mortality. Deceased patients had significantly lower hepcidin levels than survivors and were more critically ill measured by clinical evaluation scores in the ICU. No such associations were observed for hepcidin in the non-sepsis group. A negative association between hepcidin and SAPS-3 in patients with sepsis was noted at admission, suggesting that higher hepcidin values were associated with lower SAPS-3.

Jiang et al*.* reported that plasma hepcidin levels might have a predictive value, with high specificity, compared to other inflammatory anaemia-associated parameters for 28-day mortality of sepsis patients in the ICU^45^. The authors show that in a population of 198 septic patients those that survived until day 28 had an average hepcidin plasma concentration at day 1 of ≈ 53 nmol/L versus ≈ 70 nmol/L for those that did not survive^48^. These contradictory results can be explained by several differences between the present study and that of Jiang et al*.*^48^. Firstly, the studies differ in patient populations, our study consisted of a cohort of patients admitted to the ICU within 24 h after arrival to the hospital whereas Jiang et al*.* investigated a cohort that seemingly were admitted to the ICU from other hospital wards. Secondly, the patients in the present study had a 28-day mortality of 20% in the septic shock group, whereas Jiang et al*.* report a mortality of 44%. Thirdly, 69% of the septic shock patients included in the present study had bacterial infections that were confirmed by culturing, whereas Jiang et al*.* describe that only 9% of the patients had verified bacterial infections. Finally, 97% of septic shock patients in the present study needed vasopressor treatment, whereas only 5% needed vasopressor treatment in the study by Jiang et al*.*^48^. Our data on 28-day mortality is, however, in line with data on septic patients reported by Tacke et al*.*^49^.

It is possible to conclude that evaluation of serum hepcidin levels in combination with clinical scores could be beneficial for determining which patients arriving at the hospital with septic shock that may need extra supervision and other additional care.

A direct antimicrobial effect of hepcidin has been shown^19,50^ that could potentiate the antimicrobial effect of hepcidin via reduced iron concentration thereby limiting nutrition for microbes. We showed that higher hepcidin levels in serum at inclusion was associated with survival, and that hepcidin correlated negatively with SAPS 3, reflecting that a normal inflammatory response may contribute to modulating severity of illness. This is in line with experimental studies in murine models where the data show that hepcidin-induced hypoferremia can act as a defence mechanism against bacterial infection^22,51–54^. Administration of exogenous hepcidin has been suggested to be of therapeutic value in selected cases of hyperferremia^55^. High iron concentrations have been reported to increase the lethal effects of siderophile bacteria in vivo and also in fungal infections and the reduction of available plasma iron is most likely beneficial for the host unless prolonged and leading to anaemia^51,55,56^.

In the short-term perspective, it seems to be favourable for the host to reduce available iron in sepsis. A prolonged persistent iron deficiency though, may lead to harmful adverse effects for patients with a long ICU stay. This can inflict a higher risk of cognitive, and cardiovascular dysfunction, as well as higher risk of nosocomial co-infections and poor recovery after ICU treatment^56,57^. Therefore, it might be of importance to measure hepcidin repeatedly, not only in the immediate acute setting where lowered iron availability is beneficial but also at a later stage where too high levels of hepcidin might be harmful. We did not notice any increased hepcidin concentrations during the 7-day study, and no patient was discharged from the ICU with high levels of hepcidin.

The strength of the present study was that the patients were included less than 24 h after arrival to the hospital with no recent medical or surgical treatments for their present illness. Patients were not cared for by the physicians involved in the study and all patients were evaluated by available file data at the end of the study period as for having sepsis or not. Hepcidin and HBP together with conventionally used biomarkers were monitored every day for a whole week in patients suffering from sepsis compared with non-sepsis conditions. Criteria for septic shock were fulfilled in 97% of the sepsis patients and relevant microbial findings were found in the majority of these patients. The severity of illness for admittance to the ICU were similar in the two patient groups. It is well known that severe septic conditions have high in-hospital mortality rates as well as in discharged patients at later follow-ups. In this cohort we had a 180-day follow-up in mortality reflecting the negative consequences after ICU demanding care both in patients with severe sepsis and in patients with non-septic diagnosis. This is to our knowledge the first study on a mixed patient cohort that implies that hepcidin is predictive for mortality and has a protective role in vivo on survival of critically ill patients treated in the ICU.

A limitation of the study was all patients arriving to the ICU were not eligible due to the strict inclusion criteria. Any delay > 24 h before inclusion due to high workload for the ICU personnel or limited availability of beds in the ICU disqualified the patients for inclusion. The higher number of sepsis patients compared to non-sepsis patients reflects the proportions seen in the ICU. Concerning comorbidities at baseline there was an overrepresentation of cardiovascular disease and chronic kidney disease in the sepsis group which might influence the results. Previous studies have found that hepcidin levels are elevated in patients with acute coronary syndrome, in patients with type 2 diabetes mellitus, as well as in chronic kidney disease, CKD^58–60^. With the low numbers of patients with CKD in our cohort, 17 patients totally, 12 septic and 5 non-septic patients, statistics are not feasible, but median level of hepcidin was 41.5 nmol/L among the septic patients with CKD whereas only 4.7 nmol/L in the non-septic patients, the latter inconsistent with what has been reported on CKD without sepsis^59^. Hepcidin levels in the septic patients with CKD declined similarly to patients without CKD in our study indicating that sepsis was the reason for hepcidin increase and not the comorbidity. Our study included six patients with liver disease, all allocated in the sepsis group with low hepcidin values, thus any skewness would be for lower median values. A known diagnosis of liver disease would refrain the clinician from measuring hepcidin, whereas in our study these patients were initially unknown and thus included. Patients with recent surgery and blood transfusions were excluded in our cohort, since previous studies have reported markedly increased levels of hepcidin in surgical critical care, as well as in patients receiving blood transfusions that can lead to increased hepcidin levels^44,61,62^. The limited number of included patients has restricted the utility of some statistical analyses, *i.e.* adjusted logistic regressions for survival. A power calculation was not executed to estimate statistical strength of the study.

## Conclusion

The present prospective clinical study showed that increased hepcidin levels were associated with the diagnosis of septic shock versus non-septic critical illness in the ICU. The combination of hepcidin with CRP or PCT performed even better with higher sensitivity and specificity and could be helpful in diagnosing septic shock. It is an interesting possibility that the fast decline of hepcidin levels noted after initiation of treatment in septic patients could be of help for clinicians in evaluating the efficiency of treatment. We realize that additional preferentially multi-center studies are needed to substantiate this possibility. Such studies should also include hospitals with problems associated with community acquired infections with multi-resistant bacteria when decisions on antibiotic treatment is a challenge.

## Supplementary Information

Supplementary Information.

## Data Availability

The datasets used and/or analysed during the current study are available from the corresponding author on reasonable request.

## References

[1] Singer M, Deutschman CS, Seymour CW, Shankar-Hari M, Annane D, Bauer M (2016). The third international consensus definitions for sepsis and septic shock (Sepsis-3). JAMA.

[2] Paoli CJ, Reynolds MA, Sinha M, Gitlin M, Crouser E (2018). Epidemiology and costs of sepsis in the United States—an analysis based on timing of diagnosis and severity level. Crit. Care Med..

[3] Angus DC, van der Poll T (2013). Severe sepsis and septic shock. N. Engl. J. Med..

[4] Gruys E, Toussaint MJ, Niewold TA, Koopmans SJ (2005). Acute phase reaction and acute phase proteins. J. Zhejiang Univ. Sci. B.

[5] Rhodes A, Evans LE, Alhazzani W, Levy MM, Antonelli M, Ferrer R (2017). Surviving sepsis campaign: International guidelines for management of sepsis and septic shock: 2016. Crit. Care Med..

[6] Rudd KE, Johnson SC, Agesa KM, Shackelford KA, Tsoi D, Kievlan DR (2020). Global, regional, and national sepsis incidence and mortality, 1990–2017: Analysis for the Global Burden of Disease Study. Lancet.

[7] Mellhammar L (2016). Heparin-binding protein as a prognostic biomarker of sepsis and disease severity at the emergency department. Open Forum Infect Dis..

[8] Strandberg G, Walther S, Ohman CA, Lipcsey M (2020). Mortality after severe sepsis and septic shock in swedish intensive care units 2008–2016—a nationwide observational study. Acta Anaesthesiol. Scand..

[9] van Engelen TSR, Wiersinga WJ, Scicluna BP, van der Poll T (2018). Biomarkers in sepsis. Crit. Care Clin..

[10] Pierrakos C, Vincent JL (2010). Sepsis biomarkers: A review. Crit Care..

[11] Laxminarayan R, Heymann DL (2012). Challenges of drug resistance in the developing world. BMJ (Clin. Res. Ed.).

[12] Fisher J, Linder A (2017). Heparin-binding protein: A key player in the pathophysiology of organ dysfunction in sepsis. J. Intern. Med..

[13] Kandil M, Khalil G, El-Attar E, Shehata G, Hassan S (2018). Accuracy of heparin binding protein: As a new marker in prediction of acute bacterial meningitis. Braz. J. Microbiol..

[14] Shafer WM, Martin LE, Spitznagel JK (1984). Cationic antimicrobial proteins isolated from human neutrophil granulocytes in the presence of diisopropyl fluorophosphate. Infect. Immun..

[15] Linder A, Christensson B, Herwald H, Björck L, Akesson P (2009). Heparin-binding protein: An early marker of circulatory failure in sepsis. Clin. Infect. Dis..

[16] Chew MS, Linder A, Santen S, Ersson A, Herwald H, Thorlacius H (2012). Increased plasma levels of heparin-binding protein in patients with shock: A prospective, cohort study. Inflamm. Res..

[17] Wakakuri H, Hyodo H, Ohara T, Yasutake M (2019). Serum hepcidin-25 levels reflect the presence of bacteremia in patients with systemic inflammatory response syndrome. J. Nippon Med. Sch..

[18] van Eijk LT, Kroot JJ, Tromp M, van der Hoeven JG, Swinkels DW, Pickkers P (2011). Inflammation-induced hepcidin-25 is associated with the development of anemia in septic patients: An observational study. Crit. Care.

[19] Krause A, Neitz S, Mägert HJ, Schulz A, Forssmann WG, Schulz-Knappe P (2000). LEAP-1, a novel highly disulfide-bonded human peptide, exhibits antimicrobial activity. FEBS Lett..

[20] Wu TW, Tabangin M, Kusano R, Ma Y, Ridsdale R, Akinbi H (2013). The utility of serum hepcidin as a biomarker for late-onset neonatal sepsis. J. Pediatr..

[21] Kossiva L, Soldatou A, Gourgiotis DI, Stamati L, Tsentidis C (2013). Serum hepcidin: Indication of its role as an "acute phase" marker in febrile children. Ital. J. Pediatr..

[22] Scindia Y, Wlazlo E, Leeds J, Loi V, Ledesma J, Cechova S (2019). Protective role of hepcidin in polymicrobial sepsis and acute kidney injury. Front. Pharmacol..

[23] Olinder J, Ehinger D, Liljenborg E, Herwald H, Rydén C (2020). Plasma levels of hepcidin and reticulocyte haemoglobin during septic shock. J. Innate Immun..

[24] Park CH, Valore EV, Waring AJ, Ganz T (2001). Hepcidin, a urinary antimicrobial peptide synthesized in the liver. J Biol Chem..

[25] Ganz T (2013). Systemic iron homeostasis. Physiol. Rev..

[26] Nemeth E, Valore EV, Territo M, Schiller G, Lichtenstein A, Ganz T (2003). Hepcidin, a putative mediator of anemia of inflammation, is a type II acute-phase protein. Blood.

[27] Nemeth E, Rivera S, Gabayan V, Keller C, Taudorf S, Pedersen BK (2004). IL-6 mediates hypoferremia of inflammation by inducing the synthesis of the iron regulatory hormone hepcidin. J. Clin. Invest..

[28] Kemna E, Pickkers P, Nemeth E, van der Hoeven H, Swinkels D (2005). Time-course analysis of hepcidin, serum iron, and plasma cytokine levels in humans injected with LPS. Blood.

[29] Moreno RP, Metnitz PG, Almeida E, Jordan B, Bauer P, Campos RA (2005). SAPS 3—from evaluation of the patient to evaluation of the intensive care unit. Part 2: Development of a prognostic model for hospital mortality at ICU admission. Intensive Care Med..

[30] Li H, Rose MJ, Tran L, Zhang J, Miranda LP, James CA (2009). Development of a method for the sensitive and quantitative determination of hepcidin in human serum using LC–MS/MS. J. Pharmacol. Toxicol. Methods.

[31] Tapper H, Karlsson A, Morgelin M, Flodgaard H, Herwald H (2002). Secretion of heparin-binding protein from human neutrophils is determined by its localization in azurophilic granules and secretory vesicles. Blood.

[32] Galesloot TE, Vermeulen SH, Geurts-Moespot AJ, Klaver SM, Kroot JJ, van Tienoven D (2011). Serum hepcidin: Reference ranges and biochemical correlates in the general population. Blood.

[33] Itkonen O, Parkkinen J, Stenman UH, Hamalainen E (2012). Preanalytical factors and reference intervals for serum hepcidin LC–MS/MS method. Clin. Chim. Acta.

[34] Schoorl M, Snijders D, Schoorl M, Boersma WG, Bartels PC (2013). Transient impairment of reticulocyte hemoglobin content and hepcidin-25 induction in patients with community-acquired pneumonia. Scand. J. Clin. Lab. Invest..

[35] Linder A, Arnold R, Boyd JH, Zindovic M, Zindovic I, Lange A (2015). Heparin-binding protein measurement improves the prediction of severe infection with organ dysfunction in the emergency department. Crit. Care Med..

[36] Dellinger RP, Levy MM, Rhodes A, Annane D, Gerlach H, Opal SM (2013). Surviving sepsis campaign: International guidelines for management of severe sepsis and septic shock: 2012. Crit. Care Med..

[37] Pierrakos C, Velissaris D, Bisdorff M, Marshall JC, Vincent JL (2020). Biomarkers of sepsis: Time for a reappraisal. Crit. Care.

[38] Shankar-Hari M, Harrison DA, Rubenfeld GD, Rowan K (2017). Epidemiology of sepsis and septic shock in critical care units: Comparison between sepsis-2 and sepsis-3 populations using a national critical care database. Br. J. Anaesth..

[39] Garland A, Olafson K, Ramsey CD, Yogendran M, Fransoo R (2013). Epidemiology of critically ill patients in intensive care units: A population-based observational study. Crit. Care.

[40] Darveau M, Denault AY, Blais N, Notebaert E (2004). Bench-to-bedside review: Iron metabolism in critically ill patients. Crit Care..

[41] Heming N, Montravers P, Lasocki S (2011). Iron deficiency in critically ill patients: Highlighting the role of hepcidin. Crit. Care.

[42] Yeşilbaş O, Şevketoğlu E, Bursal Duramaz B, Kıhtır HS, Gedikbaşı A, Talip Petmezci M (2018). Role of hepcidin in the diagnosis of sepsis and septic shock in children. Turk. J. Med. Sci..

[43] Yan JH, Cai XY, Huang YH (2019). The clinical value of plasma hepcidin levels in predicting bacterial infections in febrile children. Pediatr. Neonatol..

[44] Cherry-Bukowiec JR, Engoren M, Wiktor A, Raghavendran K, Napolitano LM (2018). Hepcidin and anemia in surgical critical care: A prospective cohort study. Crit. Care Med..

[45] Tydén J, Herwald H, Sjöberg F, Johansson J (2016). Increased plasma levels of heparin-binding protein on admission to intensive care are associated with respiratory and circulatory failure. PLoS ONE.

[46] Tverring J, Nielsen N, Dankiewicz J, Linder A, Kahn F, Åkesson P (2020). Repeated measures of heparin-binding protein (HBP) and procalcitonin during septic shock: Biomarker kinetics and association with cardiovascular organ dysfunction. Intensive Care Med. Exp..

[47] Bergquist M, Samuelsson L, Larsson A, Tydén J, Johansson J, Lipcsey M (2020). TNFR1, TNFR2, neutrophil gelatinase-associated lipocalin and heparin binding protein in identifying sepsis and predicting outcome in an intensive care cohort. Sci. Rep..

[48] Jiang Y, Jiang FQ, Kong F, An MM, Jin BB, Cao D (2019). Inflammatory anemia-associated parameters are related to 28-day mortality in patients with sepsis admitted to the ICU: A preliminary observational study. Ann. Intensive Care.

[49] Tacke F, Nuraldeen R, Koch A, Strathmann K, Hutschenreuter G, Trautwein C (2016). Iron parameters determine the prognosis of critically Ill patients. Crit. Care Med.

[50] Maisetta G, Petruzzelli R, Brancatisano FL, Esin S, Vitali A, Campa M (2010). Antimicrobial activity of human hepcidin 20 and 25 against clinically relevant bacterial strains: Effect of copper and acidic pH. Peptides.

[51] Michels K, Nemeth E, Ganz T, Mehrad B (2015). Hepcidin and host defense against infectious diseases. PLoS Pathog..

[52] Stefanova D (2018). Hepcidin protects against lethal escherichia coli sepsis in mice inoculated with isolates from septic patients. Infect. Immun..

[53] Arezes J, Jung G, Gabayan V, Valore E, Ruchala P, Gulig PA (2015). Hepcidin-induced hypoferremia is a critical host defense mechanism against the siderophilic bacterium *Vibrio vulnificus*. Cell Host Microbe.

[54] Zeng C, Chen Q, Zhang K, Chen Q, Song S, Fang X (2015). Hepatic hepcidin protects against polymicrobial sepsis in mice by regulating host iron status. Anesthesiology.

[55] Chawla LS, Beers-Mulroy B, Tidmarsh GF (2019). Therapeutic opportunities for hepcidin in acute care medicine. Crit. Care Clin..

[56] Litton E, Lin J, Vincent J-Le (2019). Iron metabolism: An emerging therapeutic target in critical illness. Annual Update in Intensive Care and Emergency Medicine 2019/Jean-Louis Vincent.

[57] Lasocki S, Lefebvre T, Mayeur C, Puy H, Mebazaa A, Gayat E (2018). Iron deficiency diagnosed using hepcidin on critical care discharge is an independent risk factor for death and poor quality of life at one year: An observational prospective study on 1161 patients. Crit Care.

[58] Li X, Ding D, Zhang Y, Su D, Wang M, Chen X (2017). Associations of plasma hepcidin with mortality risk in patients with coronary artery disease. Oncotarget.

[59] Andrews M, Soto N, Arredondo-Olguín M (2015). Association between ferritin and hepcidin levels and inflammatory status in patients with type 2 diabetes mellitus and obesity. Nutrition.

[60] Ganz T, Nemeth E (2016). Iron balance and the role of hepcidin in chronic kidney disease. Semin. Nephrol..

[61] Pasricha SR, Frazer DM, Bowden DK, Anderson GJ (2013). Transfusion suppresses erythropoiesis and increases hepcidin in adult patients with β-thalassemia major: A longitudinal study. Blood.

[62] Lorenz L, Müller KF, Poets CF, Peter A, Olbina G, Westerman M (2015). Short-term effects of blood transfusions on hepcidin in preterm infants. Neonatology.

