# Supplementary material for: Hepcidin discriminates sepsis from other critical illness at admission to intensive care
Source: Sci Rep. 2022 Sep 1;12:14857. doi: 10.1038/s41598-022-18826-0 (PMC9434539; doi:10.1038/s41598-022-18826-0)
Supplement: Supplementary file 1 — Supplementary Information. [file 41598_2022_18826_MOESM1_ESM.docx]

Fig. 1

| Variable | Cut-off level | Sensitivity, % | Specificity, % | Positive predictive value, % | Negative predictive value, % |
| --- | --- | --- | --- | --- | --- |
| Hepcidin | ≥12.5 nmol/L | 89.7 | 49.1 | 74.4 | 72.3 |
| HBP | ≥20.1 ng/mL | 86.6 | 47.4 | 73.0 | 67.3 |
| PCT | ≥2.1 µg/L | 86.6 | 59.6 | 75.9 | 71.2 |
| CRP | ≥101.5 mg/L | 81.4 | 73.7 | 83.5 | 71.6 |
| Lactate | ≥2.4 mmol/L | 58.8 | 52.6 | 65.9 | 44.7 |
| WBC | ≥12.1 x10⁹/L | 47.4 | 54.4 | 61.5 | 39.5 |

Table 1.

Figure 1. Bacterial findings, y-axis represents total numbers of positive cultures for each bacteria.

## Table 1. Sensitivity, specificity, positive predictive value and negative predictive values in diagnosing sepsis/septic shock at admission of the biomarkers hepcidin, heparin-binding protein (HBP), procalcitonin (PCT), C-reactive protein (CRP), lactate and white blood cell (WBC).
